# Local-scale projections of coral reef futures and implications of the Paris Agreement

**DOI:** 10.1038/srep39666

**Published:** 2016-12-21

**Authors:** Ruben van Hooidonk, Jeffrey Maynard, Jerker Tamelander, Jamison Gove, Gabby Ahmadia, Laurie Raymundo, Gareth Williams, Scott F. Heron, Serge Planes

**Affiliations:** 1 NOAA Atlantic Oceanographic and Meteorological Laboratory, Ocean Chemistry and Ecosystems Division, 4301 Rickenbacker Causeway, Miami, FL 33149, USA; 2 Cooperative Institute for Marine and Atmospheric Studies, Rosenstiel School of Marine and Atmospheric Science, University of Miami, 4600 Rickenbacker Causeway, Miami, FL 33149, USA; 3 SymbioSeas and the Marine Applied Research Center, Wilmington NC, 28411, USA; 4 Laboratoire d’Excellence «CORAIL» USR 3278 CNRS–EPHE, CRIOBE, Papetoai, Moorea, Polynésie Française; 5 United Nations Environment Programme, Bangkok, Thailand; 6 Ecosystems and Oceanography Program, Pacific Islands Fisheries Science Center, 1845 Wasp Blvd Building 176, Honolulu, HI 96818, USA; 7 Oceans, World Wildlife Fund, 1250 24^th^ St., Washington, D.C. 20037, USA; 8 University of Guam Marine Laboratory, UOG Station, Mangilao, GU 96913, USA; 9 School of Ocean Sciences, Bangor University, Menai Bridge, Anglesey LL59 5AB, UK; 10 NOAA Coral Reef Watch, NESDIS Center for Satellite Applications and Research, 5830 University Research Ct., E/RA3, College Park, MD 20740, USA; 11 Global Science and Technology Inc., Greenbelt, MD 20770, USA; 12 Marine Geophysical Laboratory, Physics Department, College of Science, Technology and Engineering, James Cook University, Townsville, Qld 4814, Australia

## Abstract

Increasingly frequent severe coral bleaching is among the greatest threats to coral reefs posed by climate change. Global climate models (GCMs) project great spatial variation in the timing of annual severe bleaching (ASB) conditions; a point at which reefs are certain to change and recovery will be limited. However, previous model-resolution projections (~1 × 1°) are too coarse to inform conservation planning. To meet the need for higher-resolution projections, we generated statistically downscaled projections (4-km resolution) for all coral reefs; these projections reveal high local-scale variation in ASB. Timing of ASB varies >10 years in 71 of the 87 countries and territories with >500 km^2^ of reef area. Emissions scenario RCP4.5 represents lower emissions mid-century than will eventuate if pledges made following the 2015 Paris Climate Change Conference (COP21) become reality. These pledges do little to provide reefs with more time to adapt and acclimate prior to severe bleaching conditions occurring annually. RCP4.5 adds 11 years to the global average ASB timing when compared to RCP8.5; however, >75% of reefs still experience ASB before 2070 under RCP4.5. Coral reef futures clearly vary greatly among and within countries, indicating the projections warrant consideration in most reef areas during conservation and management planning.

Reef-building corals bleach when warmer than normal sea temperatures disrupt their mutualistic relationship with the algal symbionts, called zooxanthellae, that reside within their tissues^1^. Corals can either regain their zooxanthellae^2^ and survive or die if temperature stress persists. Currently, we are in the middle of the longest global coral bleaching event on record. Unprecedented and prolonged ocean warming triggered a global coral bleaching event that started in 2014 and may extend well into 2017^3^. The length of the event means corals in some parts of the world had no time to recover in 2014 or 2015 prior to experiencing bleaching the following year. Van Hooidonk *et al*.^4^,^5^ found that a majority of coral reefs globally are projected (at model-resolution of ~100 × 100 km) to experience annual severe bleaching (ASB) by the mid-2050’s under the emissions scenario that best characterizes current conditions, RCP8.5. 2014–2016 represents what climate models suggest may become the norm over the coming five decades; i.e. recurrent bleaching. Importantly though, great spatial variation exists in the projected timing of the onset of ASB conditions among the world’s coral reefs. This variation will be a major driver of differences in the relative (on various spatial scales) vulnerability of coral reef ecosystems to climate change.

In the IPCC’s widely adopted vulnerability assessment framework^6^, vulnerability is a function of exposure to threats and sensitivity to these threats, which yields potential impacts that are moderated by adaptive capacity^7^. Sensitivity and adaptive capacity can be collectively seen as resilience^8^; the ability of a system to maintain key functions, processes and services in the face of acute disturbances and chronic stress^9^. Coral reef managers now seek to reduce vulnerability to climate change by supporting reef resilience. Adaptive resilience-based management^10^ of reefs involves shaping human-environment interactions through management actions that reduce sensitivity to climate threats (i.e. through managing and reducing local-scale anthropogenic stress).

A key challenge for reef managers lies in deciding where to target actions to reduce anthropogenic stress. Ecosystem Based Management (EBM) is becoming increasingly sophisticated^11^. Software that combines and analyzes spatial data is increasingly accessible and used in planning for EBM to strike a balance between what can be competing conservation and development objectives^12^. For example, protecting biodiversity, providing for sustainable fisheries, and minimizing user conflicts are among the highest priorities during marine spatial planning (MSP) efforts in reef areas^13^. Incorporating spatial variation in vulnerability to climate change is frequently discussed during MSP but, as yet, is rarely operationalized^10^. This will require assessing spatial variation in the key vulnerability components–exposure and resilience–at a locally relevant scale (1–10 s of km). Global climate models (GCM) that were used to project bleaching in the past operate on much larger scales, often at a scale of 100 × 100 km. The objective of this study is to generate downscaled (4-km) projections of coral bleaching conditions (i.e. exposure to a key climate threat) and to make these publicly accessible to inform conservation planning and climate policy.

Climate model projections can be downscaled statistically or dynamically. In dynamical downscaling, GCM outputs are used to drive a higher-resolution regional numerical model, enabling simulation of local oceanic conditions in greater detail. Features that affect warming patterns, such as eddies and upwelling, can be resolved with this approach but at an exorbitant computational and financial cost. Presently, parameterized regional models do not exist for all of the world’s coral reefs. In comparison, statistical downscaling is possible now for all coral reefs and is far less resource-intensive. In the statistical downscaling undertaken here, high-resolution observations of sea surface temperature (SST) are used to adjust model baselines, annual cycles and bleaching thresholds to present-day values at a 4-km scale prior to running projections. Dynamical and statistical downscaling projections of ASB in the Caribbean are compared within van Hooidonk *et al*.^14^. In this proof-of-concept study, ASB projected using statistical downscaling closely approximated (i.e. within 10 years) projections from dynamical downscaling for the great majority of locations (>90%).

An ensemble of Coupled Model Intercomparison Project phase 5^15^ models was used to produce these downscaled projections with emphasis on emissions pathway RCP8.5, which present day emissions concentrations are currently tracking above^16^. ASB conditions refer to the start of a decade in which exceedance of 8 Degree Heating Weeks (DHWs^17^) is projected for all 10 years. One DHW is equal to 1 **°**C above the maximum monthly mean (bleaching threshold) for one week. 8 DHWs is higher than the mean optimum bleaching predictor of 6.1 DHWs for the globe^18^; i.e. at 8 DHWs we can have confidence thermal stress will be sufficiently great for bleaching to occur^5^,^19^. Results for ASB under RCP8.5 are summarized by country and territory, projections based on RCP8.5 are compared to lower greenhouse gas emission scenario RCP4.5 and we quantify the effect of emissions reductions pledges made following COP21 on projected ASB timing.

## Results and Discussion

### Projecting annual severe bleaching

**Figure 1:**
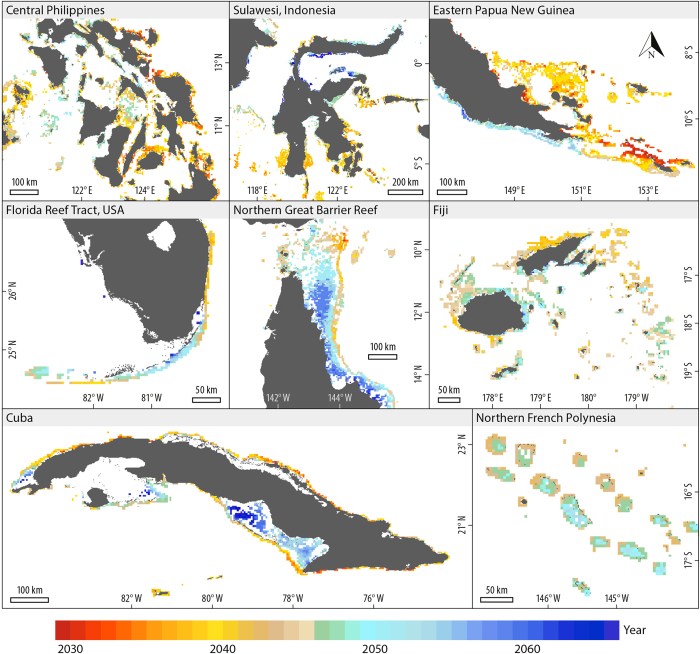
Statistically downscaled projections of the timing of the onset of annual severe bleaching (ASB) conditions under RCP8.5 for selected coral reef regions. These exemplify the high local-scale (10’s of km) variation seen in projected ASB timing in most locations and, though atypical, the low variation seen in Northern French Polynesia. This figure was created with NCL (NCAR Command Language Version 6.3.0, http://www.ncl.ucar.edu/).

Under RCP8.5, ASB is projected to occur within the 21^st^ century for 99% of the world’s coral reefs (Figs 1 and S1). The average projected year of ASB is 2043 as in ref. 5. The global range in ASB projections stretches across the entire 83-year modeled period (2089–2006). The large global range in ASB timing is driven by the 5% of reef-containing 4-km pixels (hereafter pixels) projected to experience ASB a decade or more earlier than the global average of 2043 (the relative climate losers) and the 11% projected to experience ASB a decade or more later than 2043 (the relative climate winners or ‘temporary refugia’^4^).

**Figure 2:**
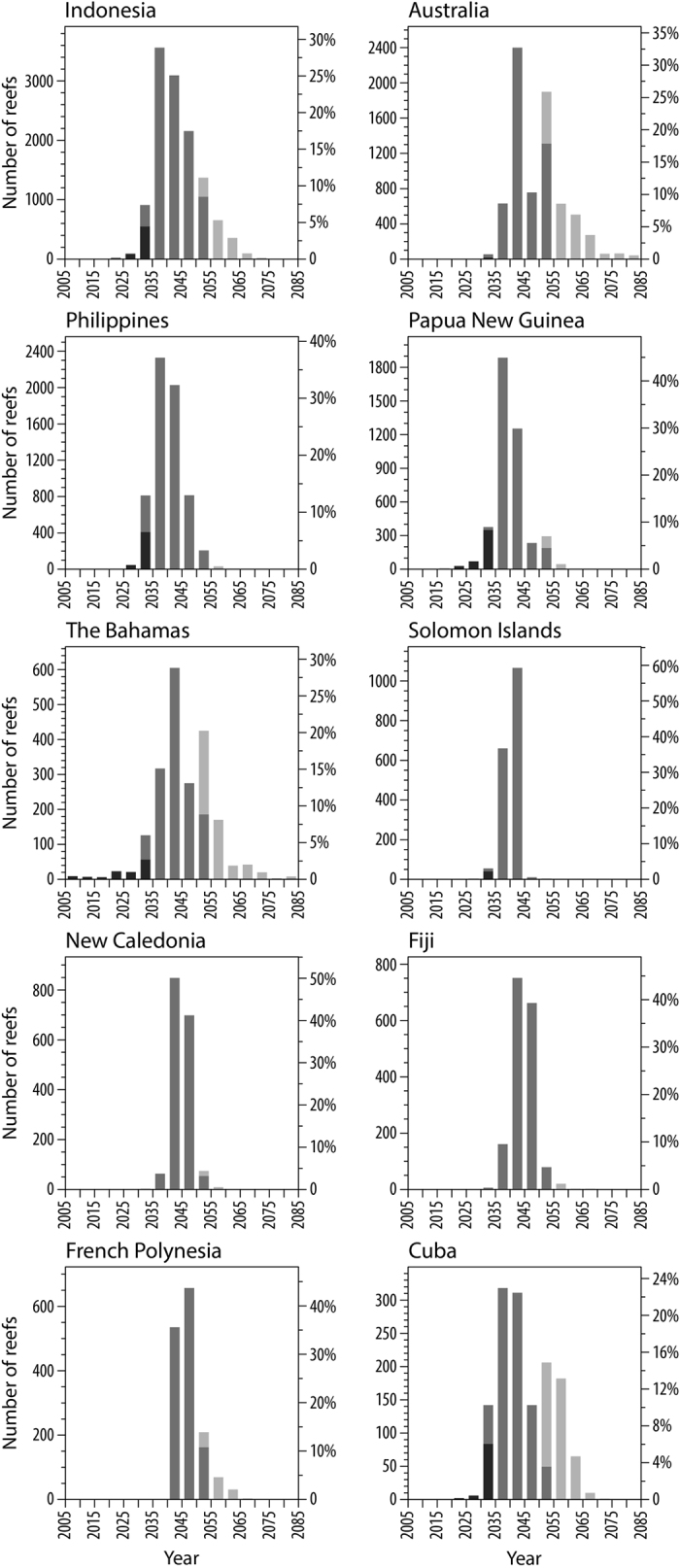
Histograms showing the distribution in projected timing of annual severe bleaching conditions under RCP8.5 for the 10 countries with the greatest reef area (see Table S1 for average years, standard deviation and range). The grey tones refer to: dark grey–relative climate losers, projected ASB before 2034, medium grey–global average of 2043 ± 10 years (2034–2053 all inclusive), light grey–relative climate winners projected ASB after 2053). This figure was created with NCL (NCAR Command Language Version 6.3.0, http://www.ncl.ucar.edu/).

Coral reef climate losers and winners occur in all of the ocean basins (Fig. 1); however, some countries have more climate winners than others. In five of the 20 countries with the greatest reef area (Table S1), >20% of the reef pixels are winners (i.e. projected ASB after 2053), including: Egypt (37%), Australia (29%), Cuba (22%), Bahamas (21%), and India (20%, Fig. 2). In five of the 20 countries with the greatest reef area, <5% of pixels are relative climate losers (i.e., projected ASB before 2033), including: Saudi Arabia (33%), Egypt (33%), Papua New Guinea (8%), Madagascar (7%), and the Bahamas (5%). The range in projected ASB timing also varies greatly among countries. Among the top 20 countries in terms of reef area, the Bahamas and Saudi Arabia have the greatest projected range in ASB timing with >80 years (equivalent to global range). Four other countries among the top 20 for reef area have a projected range in ASB timing >60 years, including: Egypt (67 yrs), Indonesia (65), Australia (64), and the United States (60). The Maldives (9 years), Federated States of Micronesia (12 years) and Marshall Islands (6 years) have the lowest projected range in ASB timing (among top 20 countries in reef area). Coral reef futures clearly vary greatly among and within countries.

**Figure 3:**
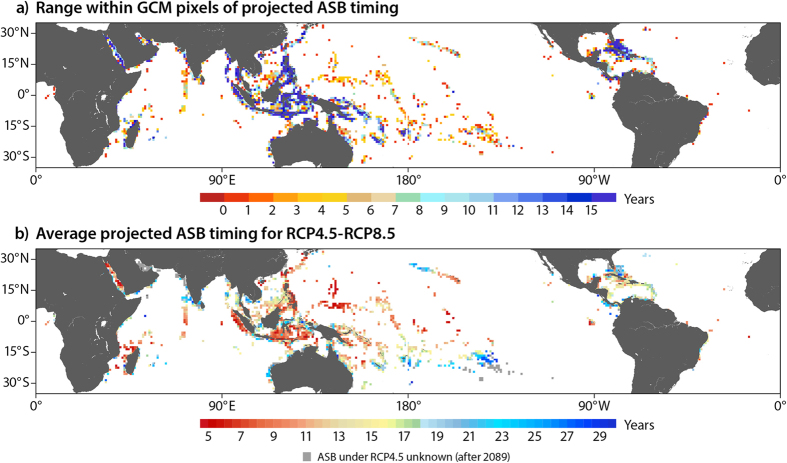
Range for statistically downscaled projections of annual severe bleaching (ASB) timing (years) at 4 km resolution within GCM pixels under RCP8.5 (**a**), and the average difference within GCM pixels between RCP4.5 and RCP8.5 in projected ASB timing (**b**). This figure was created with NCL (NCAR Command Language Version 6.3.0, http://www.ncl.ucar.edu/).

The downscaled projections can inform management decision-making where regional variation in projected ASB timing is >10 years^14^. ‘Exposure’ to the key climate threat of bleaching is sufficiently different where variation is >10 years to potentially be a driver of differences in relative vulnerability. Reefs projected to experience ASB 10 or more years later than other reefs in the same jurisdiction are *relative* refugia and are conservation priorities. Projected ASB timing varies more than 10 years on local scales (at distances of <100 km, roughly a 1° × 1° climate model pixel) in many reef areas. The extent of this local-scale variability in ASB timing can be seen at the global scale in Fig. 3a. This global map shows the range in ASB timing in the downscaled projections within the climate model pixels. 35% of the 1990 reef-containing climate model pixels have a range in ASB timing in the downscaled projections >10 years. Reefs are managed within countries and territories (i.e. model pixels are not management units), and we find that the range in projected ASB timing is >10 years in 82% (71 of 87) of the countries and territories with at least 500 km^2^ of coral reef (Table S1). In many areas there will be variation among relative refugia in anthropogenic stress. Management will have the greatest impact by targeting actions to reduce stress to relative refugia where there is local anthropogenic stress (such as land based sources of pollution, over fishing or anchor damage) that is amenable to management influence, recommended in ref. 20. This strategy of managing the ‘weak of the strong’ maximizes the likelihood that at least some reefs will be healthy in the future^21^ and continue to provide ecosystem goods and services.

In some locations, projected ASB timing varies >20 years on scales <20 km. There is an inshore-offshore gradient in the northern Great Barrier Reef, Australia, with offshore locations projected to experience ASB 15–25 years earlier than inshore locations (Fig. 1). This increases the impetus to reduce anthropogenic stress at inshore locations where, for example, efforts to reduce land-based sources of pollution and improve water quality will have greater impact anyway^22^.There is an east-west gradient in eastern Papua New Guinea (PNG); islands to the east are projected to experience ASB >25 years earlier than reefs to the west on the southern side of PNG. A similar gradient is seen in the Philippines with eastern sides of the eastern islands projected to experience ASB >20 years earlier than central islands. The Philippines and eastern PNG results are likely explained by the eastern sides of these areas having greater exposure to the western Pacific warm pool, which has higher warming rates than SE Asia^23^. Though atypical, there are also countries/territories (with >400 km^2^ of reef area) where variation in the projected ASB timing is <10 years. These are in the south Pacific (French Polynesia) and within the western Pacific warm pool (Tuvalu [ASB average and range–2039, 9], Tokelau [2039, 4], and the Marshall Islands [2040, 6]).

The high local-scale variation in projected ASB timing is due to variation in the difference between typical warm season temperatures and the maximum monthly mean (MMM). The MMM is the warmest month in the climatology (1982–2008) and is the threshold used to determine when temperatures become stressful to corals and cause bleaching^14^,^24^. Typical warm season temperatures in some locations are closer to MMM than typical temperatures at locations only 10’s of km away and these locations are projected to experience ASB sooner (and vice versa). Statistically downscaling the model-resolution projections ensures local-scale variation in differences between typical warm season temperatures and MMM are accounted for; i.e. rather than smoothed over through averaging, as is the case in the numerous studies that present model-resolution projections^4^,^5^,^25^,^26^,^27^,^28^,^29^. However, these results should be interpreted with caution where currents are expected to shift under climate change (e.g. Pacific equatorial undercurrent^25^, (PEU) or the Loop current (LC) in the Gulf of Mexico and western Caribbean^26^). In these locations, warm water residence times over reefs may decrease (PEU) or increase (LC) during warm seasons as the currents shift. Future research can include further development of dynamically downscaled projections of coral bleaching conditions, as regional ocean models become available and are parameterized.

### Climate policy implications for coral bleaching

The UN Framework on Climate Change Convention (UNFCCC) now has a near-universal membership of 195 countries, aimed at stabilizing atmospheric concentrations of greenhouse gases to avoid “dangerous anthropogenic interference with the climate system” (UNFCCC article 2, http://unfccc.int/key_documents/the_convention/items/2853.php). The membership meets annually at a Conference of Parties (COP) to review the Convention’s implementation. COP21, held in Paris in 2015, adopted a legally binding agreement with the goal of keeping global warming below 2 °C and potentially to 1.5 °C (‘the Paris Agreement’). As of September, 2016, 162 countries have submitted Intended Nationally Determined Contributions (INDCs) in pursuit of this goal. The countries pledging to reduce greenhouse gases account for >90% of global emissions. New INDCs are expected in 2020 and then every five years thereafter with global stock-take to review progress planned for 2023.

If the COP21 pledges never become reality, 58-61 Gtons CO_2_-eq/yr are projected to be emitted in 2030 (http://climateactiontracker.org/global.html), which is closely approximated by emissions scenario RCP8.5. Assuming pledges do become reality would result in 52-55 Gtons CO_2_-eq/yr in 2030, which is below RCP8.5 and above the emissions concentrations associated with RCP4.5. Pledges made under the recent INDCs would have to be 150% greater on average for emissions in 2030 to be 49 Gtons CO_2_-eq/yr, which is the prescribed amount under the RCP4.5 scenario. RCP4.5 represents a better future for coral reefs than would be projected using the emissions trajectory associated with the COP21 pledges. We compare projections for RCP4.5 and RCP8.5, and discuss implications for coral reefs of CO_2_ emissions reductions that are ~1.5 times that pledged under COP21 as of April, 2016.

**Figure 4:**
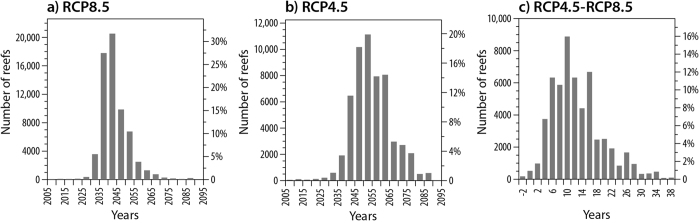
Histograms showing the distribution in projected timing of annual severe bleaching conditions under two emissions scenarios (RCP 8.5 and RCP 4.5; **a** and **b**). The difference between these scenarios is shown in (**c**) for the 86% of reefs for which ASB is projected this century under both RCP8.5 and RCP4.5. This figure was created with NCL (NCAR Command Language Version 6.3.0, http://www.ncl.ucar.edu/).

The average year for the projected timing of ASB under RCP4.5 is 2054, 11 years later than the average projected under RCP8.5 (Fig. 4). ASB under RCP4.5 is projected to be >25 years later than under RCP8.5 for very few reefs (7%), and <10 years later for many reefs (32%). There is great spatial variation across the globe in the differences in projected ASB timing between RCP4.5 and 8.5 and a latitudinal gradient can be seen (Fig. 3b). Many high-latitude reefs in Australia, the south Pacific, Northwestern Hawaiian Islands, India, and the Florida Reef Tract have >25 more years before ASB occurs under RCP4.5 than 8.5. Low latitude locations in SE Asia, the Coral Triangle, and the eastern Pacific also have >25 more years before ASB occurs under RCP4.5 (Fig. 3b). In contrast, there is <10 years difference between RCP4.5 and 8.5 for reefs near the equator that are projected to experience ASB relatively early. In summary, these projections suggest that the INDCs submitted as of April, 2016 will do little to provide reefs with more time to adapt and acclimate prior to severe bleaching conditions occurring annually.

### Use of the projections in management and policy

We identify four applications for these downscaled projections, in influencing and guiding research, conservation and management planning and policy. (1) Future research projects can incorporate these data and, for example, include spatial variation in adaptive capacity when more is known about spatial variation in the ability of corals to adapt to increasing sea temperatures. (2) In conservation planning, these downscaled projections form a local-scale view of the exposure component of vulnerability. Data can also be generated on spatial variation in the resilience component of vulnerability through field-based assessments of relative resilience potential and anthropogenic stress as in ref. 30. By combining projected future exposure and resilience data, managers can map relative vulnerability to climate change and identify and prioritize actions to reduce anthropogenic stress where these will most positively influence site and system resilience^10^,^30^. (3) In support of (2), managers and conservationists can use the downscaled projections in outreach campaigns to raise awareness and educate about climate change as well as explain and support planned actions to reduce climate vulnerability.

(4) The downscaled projections can also inform policy decisions. The pledges that followed COP21 are a great step forward. However, much greater emissions reductions are required to prevent the great majority of coral reefs from experiencing severe bleaching conditions annually within this century. This reinforces the importance of pursuing efforts to limit the temperature increase to 1.5 degrees Celsius. This is especially the case given the widespread coral bleaching that has been occurring globally since 2014 with global warming of 0.9 °C. This recent bleaching event and the findings presented here deserve attention in policy discussions at national and international levels, including during the development of INDCs in 2020. The downscaled projections can also be used to assess both socio-economic and ecological vulnerability to coral bleaching and inform policies related to fisheries, coastal development and conservation, and land-use planning including agriculture, all of which influence reef resilience and vulnerability^31^,^32^. Downscaled projections could also play a role in planning management actions aimed at reducing dependence on reefs; such actions may both reduce local stressors on reefs and reduce the socio-economic sensitivity of coastal communities. Using these downscaled projections in all four of the identified ways can support efforts to reduce the vulnerability of coral reefs to climate change.

## Methods

Sea surface temperature (SST) data from GCMs was obtained from the CMIP5 for the RCP8.5 and RCP4.5 experiments^33^. For the statistical downscaling, model outputs from 33 GCMs for RCP8.5 and 35 GCMs for RCP4.5 were adjusted as follows see ref. 4 for models list. The annual cycle in the models was substituted with a bi-linear interpolation of the observed mean and annual cycle from 1982–2008 from the NOAA Pathfinder v5.0 climatology, which has a 4-km resolution^34^. Where model outputs were missing due to a land mask, SSTs were interpolated in the zonal direction. The Pathfinder SST archive is used for climate model adjustment as it is the U.S. official climate record for SST. Reasons the Pathfinder v5.0 climatology is used are reviewed in van Hooidonk *et al*.^14^. The 1982–2008 period is slightly cooler than the 2006-2011 period. This makes our projections slightly conservative or ‘optimistic’; i.e., the bleaching conditions we project could occur earlier than the projected date. After interpolating the model data to the 4 km Pathfinder grid, the mean for 2006–2011 was replaced for each 4-km pixel with the mean of the climatology at that location. These steps ensure: (1) the climate models are adjusted to observed data as in refs 4,5,14 and 35, and (2) that these downscaled 4-km projections have a locally relevant SST starting point from which the climate models then project future SST.

Degree heating months were calculated for each year between 2006 and 2099 as anomalies above the warmest monthly temperature (the maximum monthly mean or MMM) from the Pathfinder climatology^34^ and were summed for each period of 3 consecutive months in the time series. A review of climatological thresholds from which bleaching stress can be calculated can be found in van Hooidonk *et al*.^14^, which includes defense of the use of MMM. Degree heating months are converted into DHW by multiplying by 4.35^5^,^14^, as in ref. 36. The onset of annual severe coral bleaching (ASB) is defined as the annual exceedance of >8 DHW accumulating during any 3-month period (as in refs 5 and 14). The focus here is on spatiotemporal variability in the projections. Hypothetical adaptation rates are not applied^36^,^37^,^38^ here. Applying the same adaptation rates everywhere is not well founded; spatial variation in adaptive capacity is certainly great and is largely unknown. Adding in the same adaptation rate everywhere shifts projected bleaching timing back, yet the projected spatial patterns do not change and these should be the focus for local management planning.

In comparing the projected timing of the onset of ASB between RCP8.5 and 4.5, the dates projected for 8.5 were subtracted from 4.5. This yielded the amount of additional time coral reefs will have (in most cases) or not (in rare cases) to adapt or acclimate to increasing temperatures if emissions cuts are made that are in keeping with RCP4.5 (e.g. ~1.5x the COP21 pledges). We calculate the range within climate model pixels for ASB under RCP8.5 (latest year minus earliest year). To examine model agreement, we present the standard deviation of the projections for ASB timing from among the models used for RCP8.5. Our reef locations dataset was classified into countries and territories using EEZ boundaries from www.marineregions.org, which we then cross-checked with UNEP dataset of global distribution of coral reefs (downloaded January 2016, http://data.unep-wcmc.org/datasets/1).

The onset of annual bleaching conditions is defined as the annual exceedance of >8 DHW accumulating during any 3-month period. All downscaled projections of ASB from the models were averaged to create a multi model ensemble. The number of models used, normal distribution of model outputs for most pixels, and absence of many outliers all support use of the mean over the median^39^. This is a common approach and was done to reduce unknown, sometimes time-dependent errors in the models^40^. Further, it is not possible to robustly examine model performance (regionally or globally) in hindcasting DHWs to predict past bleaching events due to the paucity of bleaching and non-bleaching observations data. For that reason, we include all models and do not attach different weights to models before creating an ensemble see also^4^,^5^,^14^,^35^. All model outputs were reduced to a subset of only reef locations obtained by combining three published global reef-locations datasets ReefBase (http://www.reefbase.org), Millennium Coral Reef Mapping Project (http://imars.usf.edu/MC/), and Reefs at Risk-Revisited (http://www.wri.org/publication/reefs-risk-revisited). This was further augmented by other coral reef locations documented by this author group and colleagues, especially in the US-affiliated Pacific islands; the reef locations dataset (4-km resolution) is available at http://coralreefwatch.noaa.gov. The numbers of 4-km pixels containing reefs within each climate model pixel is shown in Figure S3 and varies from 1–391.

To facilitate use of the projections, the results for RCP8.5 and RCP4.5 are presented within Google Earth files and made publicly accessible via the UNEP-Live (http://www.uneplive.unep.org) and NOAA Coral Reef Watch websites (http://coralreefwatch.noaa.gov/climate/projections/downscaled_bleaching_4km/index.php). All interpolation was performed using Climate Data Operators, a software package available from: https://code.zmaw.de/projects/cdo/. All model output adjustment, projections, data visualization and analysis were conducted using the NCAR Command Language (NCL; http://www.ncl.ucar.edu/).

## Additional Information

**How to cite this article**: van Hooidonk, R. *et al*. Local-scale projections of coral reef futures and implications of the Paris Agreement. *Sci. Rep.* **6**, 39666; doi: 10.1038/srep39666 (2016).

**Publisher's note:** Springer Nature remains neutral with regard to jurisdictional claims in published maps and institutional affiliations.

## Supplementary Material

Supplementary Information
