# Supplementary material for: Local-scale projections of coral reef futures and implications of the Paris Agreement
Source: Sci Rep. 2016 Dec 21;6:39666. doi: 10.1038/srep39666 (PMC5175274; doi:10.1038/srep39666)
Supplement: Supplementary Information [file srep39666-s1.zip › Figure_S1.pdf]

Local-scale projections of coral reef futures and implications of the Paris Agreement

Ruben van Hooidonk, Jeffrey Maynard, Jerker Tamelander, Jamison Gove, Gabby Ahmadia, Laurie Raymundo, Gareth Williams, Scott Heron1, Serge Planes

Figure S1. Separate supplementary material file. 30,000 x 30,000 pixel image shows projected timing of the onset of annual severe bleaching under RCP8.5 at 4-km resolution. This figure was created with NCL (NCAR Command Language Version 6.3.0, http://www.ncl.ucar.edu/).

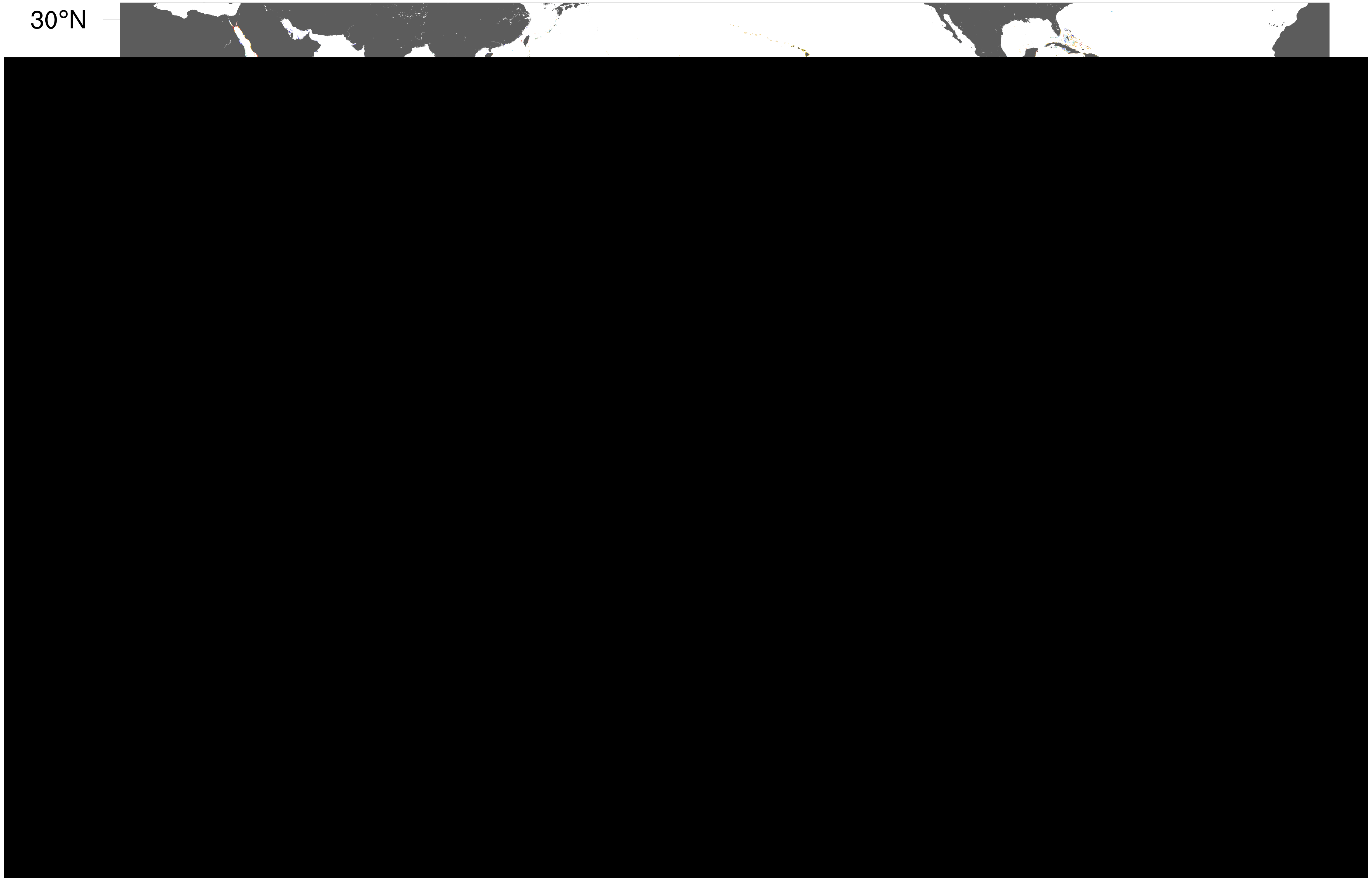
30°N
