# Supplementary material for: Local-scale projections of coral reef futures and implications of the Paris Agreement
Source: Sci Rep. 2016 Dec 21;6:39666. doi: 10.1038/srep39666 (PMC5175274; doi:10.1038/srep39666)
Supplement: Supplementary Information [file srep39666-s1.zip › Local-scale projections_SM_Rev3.pdf]

Local-scale projections of coral reef futures and implications of the Paris Agreement

Ruben van Hooidonk[1, 2*], Jeffrey Maynard[3, 4], Jerker Tamelander[5], Jamison Gove[6], Gabriella Ahmadia[7], Laurie Raymundo[8], Gareth Williams[9], Scott Heron[10, 11], Serge Planes[4]

*Corresponding Author. E-mail address: ruben.van.hooidonk@noaa.gov

Supplementary material

Table S1. Summaries for countries and territories of the number of 4-km reef-containing pixels, range in the projected timing of the onset of annual severe bleaching (ASB) under RCP8.5 across pixels, the average projected year for ASB and standard deviation around the average. Reef-containing pixels are linked to countries and territories by overlaying EEZ boundaries from www.marineregions.org, which we then cross-checked with UNEP dataset of global distribution of coral reefs (downloaded January 2016, http://data.unep-wcmc.org/datasets/1). The top 20 countries in terms of reef are listed first from most to least area; all other countries and territories are listed alphabetically.

Country/Territory	Number pixels	Range	Average year	STDEV
**Indonesia**	12340	65	2044	7.61
**Australia**	7334	64	2050	8.82
**Philippines**	6274	44	2040	4.76
**Papua New Guinea**	4197	50	2040	5.90
**The Bahamas**	2100	81	2046	9.63
**Solomon Islands**	1798	44	2040	2.81
**New Caledonia**	1694	22	2044	3.39
**Fiji**	1685	41	2044	4.04
**French Polynesia**	1504	25	2047	4.44
**United States**	1417	60	2042	4.80
**Cuba**	1385	48	2045	8.85
**Saudi Arabia**	1311	82	2037	10.82
**Maldives**	1207	9	2041	1.93
**Micronesia**	1096	12	2038	1.46
**Madagascar**	1006	42	2044	6.72
**Marshall Islands**	952	6	2040	1.50

Country/Territory	Number pixels	Range	Average year	STDEV
**India**	936	56	2049	8.60
**Egypt**	843	67	2041	18.50
**Malaysia**	829	30	2041	6.10
**Spratly Islands**	699	8	2042	1.70
**American Samoa**	77	9	2039	1.64
**Anguilla**	19	9	2045	2.31
**Antigua & Barbuda**	44	10	2044	3.20
**Argentina**	1	0	2062	0
**Aruba**	19	3	2042	1.32
**Bahrain**	73	25	2084	4.45
**Bangladesh**	5	6	2049	2.19
**Barbados**	27	9	2046	1.80
**Belize**	334	20	2040	4.33
**Bermuda**	70	16	2050	3.77
**Bonaire; Sint-Eustasius; Saba**	55	7	2044	2.12
**Brazil**	349	24	2043	4.82
**British Indian Ocean Territory (including Chagos)**	569	7	2041	1.97
**British Virgin Islands**	59	9	2044	2.87
**Brunei**	37	14	2048	3.19
**Cambodia**	59	23	2051	5.42
**Cameroon**	1	0	2055	0
**Cape Verde**	1	0	2045	0
**Cayman Islands**	43	7	2039	1.85
**Chile**	1	0	2069	0
**China**	180	81	2057	17.67
**Christmas Islands**	11	10	2041	3.22

Country/Territory	Number pixels	Range	Average year	STDEV
**Clipperton Island**	4	4	2041	2.31
**Cocos Island**	23	5	2043	1.60
**Colombia**	189	43	2043	5.28
**Comoros**	104	14	2042	2.42
**Cook Islands**	128	15	2044	3.99
**Costa Rica**	77	28	2049	5.90
**Curaçao**	39	4	2041	1.32
**Djibouti**	84	15	2046	3.87
**Dominica**	27	6	2045	1.80
**Dominican Republic**	273	28	2041	3.67
**Ecuador**	86	21	2042	4.14
**El Salvador**	2	0	2050	0
**Equatorial Guinea**	3	8	2044	4.36
**Eritrea**	503	53	2053	10.86
**Ethiopia**	10	7	2038	2.36
**French Guiana**	1	0	2038	0
**French Southern & Antarctic Lands**	62	9	2041	2.45
**Grenada**	38	12	2045	2.37
**Guadeloupe**	78	9	2046	1.73
**Haiti**	313	23	2040	3.41
**Honduras**	226	20	2042	3.45
**Iran**	107	21	2068	5.27
**Israel**	4	22	2017	9.95
**Jamaica**	223	14	2040	2.42
**Japan**	532	27	2047	4.30
**Jordan**	4	4	2012	2.00
**Kenya**	159	24	2047	3.28

Country/Territory	Number pixels	Range	Average year	STDEV
**Kiribati**	93	14	2041	3.50
**Kuwait**	5	5	2086	2.12
**Martinique**	52	6	2046	1.29
**Mauritius**	150	21	2046	3.93
**Mayotte**	106	12	2041	2.32
**Mexico**	371	62	2039	8.44
**Montserrat**	8	7	2042	2.27
**Mozambique**	461	35	2040	5.91
**Myanmar**	539	46	2046	7.12
**Nauru**	6	0	2035	0.00
**New Zealand**	4	10	2047	4.72
**Nicaragua**	278	14	2038	2.52
**Niue**	16	19	2047	6.36
**Northern Marinana Islands & Guam**	271	12	2037	2.20
**Oman**	192	37	2055	8.17
**Palau**	188	11	2038	2.41
**Panama**	348	42	2045	6.96
**Paracel Islands**	104	7	2037	1.82
**Pitcairn Islands**	18	13	2059	2.99
**Protected zone Australia/Papua New Guinea**	16	7	2043	1.94
**Puerto Rico**	182	17	2042	3.06
**Qatar**	45	21	2077	8.24
**Reunion**	13	11	2045	3.01
**Saint Helena; Ascension en Tristan da Cunha**	1	0	2043	0
**Saint Martin**	17	11	2041	3.19
**Saint Vincent and the Grenadines**	29	3	2046	1.05

Country/Territory	Number pixels	Range	Average year	STDEV
**Samoa**	116	9	2038	1.80
**Sao Tome & Principe**	1	0	2043	0
**Seychelles**	308	19	2042	3.46
**Singapore**	13	4	2044	1.09
**Sint Maarten**	3	3	2045	1.73
**Somalia**	153	25	2048	4.17
**South Africa**	18	36	2051	7.75
**Sri Lanka**	131	29	2050	5.91
**St. Kitts & Nevis**	27	8	2043	2.95
**St. Lucia**	28	9	2045	2.30
**Sudan**	257	23	2049	3.81
**Taiwan**	164	58	2037	12.35
**Tanzania**	635	45	2041	4.04
**Thailand**	349	36	2046	4.12
**Timor-Leste**	78	20	2040	5.34
**Tokelau**	26	4	2039	0.82
**Tonga**	396	22	2045	2.51
**Trinidad & Tobago**	34	19	2047	3.52
**Turks & Caicos Islands**	117	18	2036	3.68
**Tuvalu**	217	9	2039	1.87
**United Arab Emirates**	98	79	2079	11.64
**United States Minor Outlying Islands**	461	21	2039	2.94
**United States Virgin Islands**	62	7	2044	2.66
**Vanuatu**	601	21	2043	2.97
**Venezuela**	193	33	2047	6.79
**Vietnam**	322	55	2048	12.93
**Wallis & Futuna**	133	10	2038	1.45

Country/Territory	Number pixels	Range	Average year	STDEV
**Yemen**	312	28	2045	5.94

Table S2. Numbers of reef pixels and percentages for the histograms shown within Figure 4. The histograms in Figure 4 show the distribution in projected timing of annual severe bleaching conditions under the two emissions scenarios (a and b). The difference between these scenarios is shown in (c) for the 86% of reefs for which ASB is projected this century under both RCP8.5 and RCP4.5.

Year	RCP8.5 (N pixels)	RCP8.5 (%)	RCP4.5 (N pixels)	RCP4.5 (%)	Years Difference	RCP4.5-RCP8.5 (N pixels)	RCP4.5-RCP8.5 (%)
**2005**	73	0.11	66	0.12	**-2**	175	0.31
**2010**	107	0.17	117	0.21	**0**	520	0.94
**2015**	82	0.13	76	0.14	**2**	976	1.76
**2020**	125	0.19	142	0.26	**4**	3749	6.74
**2025**	515	0.80	223	0.40	**6**	6328	11.38
**2030**	3599	5.56	590	1.06	**8**	5873	10.56
**2035**	17829	27.53	1922	3.45	**10**	8882	15.98
**2040**	20533	31.71	6459	11.60	**12**	6326	11.38
**2045**	9865	15.23	10146	18.23	**14**	4415	7.94
**2050**	6762	10.44	11098	19.94	**16**	6675	12.01
**2055**	2501	3.86	7921	14.23	**18**	2463	4.43
**2060**	1300	2.01	8043	14.45	**20**	2510	4.51
**2065**	742	1.15	2966	5.33	**22**	1914	3.44
**2070**	269	0.42	2715	4.88	**24**	846	1.52
**2075**	164	0.25	2085	3.75	**26**	1664	2.99
**2080**	79	0.12	510	0.92	**28**	952	1.71
**2085**	210	0.32	579	1.04	**30**	331	0.60
**2090**	0	0.00	0	0.00	**32**	355	0.64
					**34**	467	0.84
					**36**	76	0.14
					**38**	99	0.18

Figure S1. Separate supplementary material file in PDF form. 30,000 x 30,000 pixel image shows projected timing of the onset of annual severe bleaching under RCP8.5 at 4-km resolution. This figure was created with NCL (NCAR Command Language Version 6.3.0, http://www.ncl.ucar.edu/).

Figure S2. Separate supplementary material file in PDF form. 30,000 x 30,000 pixel image shows projected timing of the onset of annual severe bleaching under RCP4.5 at 4-km resolution. This figure was created with NCL (NCAR Command Language Version 6.3.0, http://www.ncl.ucar.edu/).

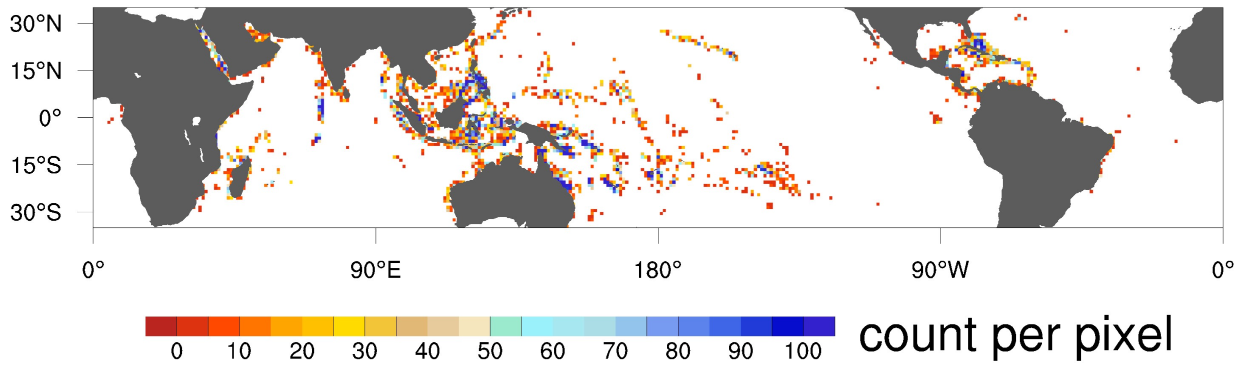

Figure S3. Number of 4-km reef-containing pixels within the GCM data grid from which the downscaled projections were produced. This GCM data grid is used to visualize global-scale patterns in Figure 1. This figure was created with NCL (NCAR Command Language Version 6.3.0, http://www.ncl.ucar.edu/).
